# Evaluation of Glycogen Storage Patients: Report of Twelve Novel Variants and New Clinical Findings in a Turkish Population

**DOI:** 10.3390/genes12121987

**Published:** 2021-12-15

**Authors:** Melike Ersoy, Bulent Uyanik, Asuman Gedikbasi

**Affiliations:** 1 Division of Pediatric Metabolic Diseases, Department of Pediatrics, Bakirkoy Dr. Sadi Konuk Training and Research Hospital, University of Health Sciences, Istanbul 34093, Turkey; melike.ersoy@saglik.gov.tr; 2 Division of Medical Genetics, Department of Internal Medicine, Faculty of Medicine, Bezmialem Foundation University, Istanbul 34093, Turkey; buyanik@bezmialem.edu.tr; 3 Division of Medical Genetics, Department of Pediatric Basic Sciences, Institute of Child Health, Istanbul University, Istanbul 34104, Turkey

**Keywords:** glycogen storage disease, genotype–phenotype, novel variants, new clinic findings

## Abstract

Glycogen storage diseases (GSDs) are clinically and genetically heterogeneous disorders that disturb glycogen synthesis or utilization. Although it is one of the oldest inherited metabolic disorders, new genetic methods and long-time patient follow-ups provide us with unique insight into the genotype–phenotype correlations. The aim of this study was to share the phenotypic features and molecular diagnostic results that include new pathogenic variants in our GSD cases. Twenty-six GSD patients were evaluated retrospectively. Demographic data, initial laboratory and imaging features, and current findings of the patients were recorded. Molecular analysis results were classified as novel or previously defined variants. Novel variants were analyzed with pathogenicity prediction tools according to American College of Medical Genetics and Genomics (ACGM) criteria. Twelve novel and rare variants in six different genes were associated with the disease. Hearing impairment in two patients with GSD I, early peripheral neuropathy after liver transplantation in one patient with GSD IV, epilepsy and neuromotor retardation in three patients with GSD IXA were determined. We characterized a heterogeneous group of all diagnosed GSDs over a 5-year period in our institution, and identified novel variants and new clinical findings. It is still difficult to establish a genotype–phenotype correlation in GSDs.

## 1. Introduction

Glycogen storage diseases [GSDs] are a large group of inherited metabolic diseases with abnormal storage or utilization of glycogen. They affect primarily the liver and muscle, followed by the nervous system, kidneys, intestine, and leukocytes [1]. The incidence of all forms of glycogen storage disease is 1/10,000 [2]. Depending on the type of enzyme deficiency in tissue, it is classified as muscle or liver glycogenosis. However, both muscle and liver can be affected in some types [3]. The diagnosis of GSDs is based on the enzyme assay and/or molecular analysis as a result of the biochemical analysis and biopsy examination of the patients with characteristic signs [4]. GSDs have broad genetic heterogeneity and phenotypical variations. Some GSDs lead to death within the first years of life, whereas some remain asymptomatic for life. Genotype–phenotype correlation has been reported for some mutations in GSD patients [5]. Patients with late-onset or atypical presentation can experience a delay in diagnosis and proper treatment. Some unexpected clinical findings may accompany classical features [6]. The aim of this study was to characterize the phenotype of the followed GSDs over a 5-year period in our center, and to report novel variants. Thus, we contributed to knowledge on the phenotype–genotype correlation by reporting the new clinical findings we identified.

## 2. Materials and Methods

The initial clinical and biochemical findings of the patients diagnosed in metabolism center of Bakirkoy Dr. Sadi Konuk Training and Research Hospital between January 2015 and June 2020 were evaluated retrospectively as a cross-sectional study. The study protocol was approved by the Institutional Ethics Committee (approved number: 2020/66; date: 22 March 2020). Written informed consent was obtained from all legal guardians before study enrollment.

Biochemical, clinical, and imaging data of the patients were obtained from the hospital electronic system and patient files. High-purity DNA was isolated from peripheral blood leukocytes using a DNA isolation kit (PureLink™ Genomic DNA Mini Kit, Invitrogen, CA, USA) following the manufacturer’s protocol, in all patients. In order to elucidate the molecular etiology, two massive parallel-sequencing methods were used to identify variants that cause GSDs; targeted gene panel and clinical exome sequencing (CES). Twenty patients were examined by targeted gene-panel sequencing involving the use of a customized panel including 16 GSD-associated genes (*GYS2*, *GYS1*, *G6PC*, *SLC37A4*, *AGL*, *GBE1*, *PYGM*, *PYGL*, *PFKM*, *PHKA2*, *PHKB*, *PHKG2*, *PHKA1*, *PGAM2*, and *PGM1*). Six patients with unexplained liver and muscle enzyme elevation or neuromotor retardation were examined by Clinical Exome Sequencing (CES) using the Illumina Clinical-Exome Sequencing TruSight One Gene Panel. In both the panel and the CES, the libraries generated were sequenced with 250-bp paired-end reads using the Illumina MiSeq or Nextseq500 next-generation sequencing platforms. Detected variants were confirmed by conventional Sanger sequencing using the BigDye Terminator Cycle Sequencing kit (Applied Biosystems, Foster City, CA, USA) using both the patients’ genomic DNA, and if available, that of their parents.

## 3. Results

### 3.1. Demographic and Laboratory Findings

Twenty-six patients diagnosed with GSD from 24 unrelated families were investigated. Demographic data of the patients are summarized in Table 1. The initial laboratory findings are shown in Table 2. As a result of molecular analysis, 12 novel pathogenic variations were detected; *GYS2* c.607A>G (p.Thr203Ala) and c.1307A>C (p.Gln436Pro), *G6PC* c.562+1G>A (p.?), *GBE1* c.1054G>C (p.Asp352His), *PYGL* c.1355G>T (p.Gly452Val), c.2380-1G>C p.(?), c.921_924del (p.His308Leufs*8), *PHKA2* c.1978C>T (p.Leu660Phe), c.3028-2A>G (p.?), c.3201G>T (p.Trp1067Cys), *PHKB* exon 18–21 deletion (p.?), *PHKA1* c.1963C>T (p.Arg655Cys) (Table 3).

### 3.2. Clinical Findings

- P1-P3-GSD 0: P1 and P2 (two siblings) had symptomatic hypoglycemia, whereas P3 had asymptomatic hypoglycemia The mean age at diagnosis was 15.67 ± 23.33 months.
- P4-P9-GSDI: All six patients had typical clinical and laboratory features (hepatomegaly, hypoglycemia, lactic acidosis, hyperuricemia, hypercholesterolemia, and hypertriglyceridemia, and neutropenia for GSDIb-P9). Four patients had severe osteoporosis (P4, P6, P7, P8). Three patients (P6, P7, P8) had renal involvement including parenchymal heterogeneity and enlargement in kidneys with microalbuminuria despite good metabolic control. None of the patients developed chronic renal failure. Dialysis and transplantation were not required. Two (P6, P7) had nonfamilial sensorineural-type hearing loss. Congenital hypothyroidism was found in one GSDI patient (P5) (Table 1). The mean age at diagnosis was 2 ± 1.26 months.
- P10-P12-GSD III: Three patients were presented with typical signs of hypoglycemia, myopathy, and hepatopathy. Short stature developed only in P12. The mean age at diagnosis was 10.3 ± 7.63 months.
- P13-P14-GSD Type IV: Both patients underwent living donor liver transplantation at the age of 3 and 2 years, respectively, due to severe liver failure. Persistent mild neuromotor retardation and peripheral sensory neuropathy were detected after transplantation in P13. The mean age at diagnosis was 15.5 ± 9.19 months.
- P15-P16-GSD Type V: Both patients had mild muscle enzyme elevation with muscle pain and fatigue. None of them had any rhabdomyolysis attacks. The mean age at diagnosis was 26.5 ± 2.12 months.
- P17-P20-GSD Type VI: All patients presented with mild-to-moderate hepatomegaly, short stature, and ketotic hypoglycemia. The mean age at diagnosis was 6 ± 1.23 months.
- P21-26-GSD Type IX: All patients had hepatic involvement. Additionally, short stature was detected in three (P21, P22, P23), and hypertrophic cardiomyopathy was detected in one patient (P23). Psychomotor retardation was prominent in three patients (P22, P23, P24). P22 and P24 had epilepsy. Autism spectrum findings were determined in P22. The mean age at diagnosis was 15 ± 6.54 months.

## 4. Discussion

We presented the clinical features and results of molecular analysis of 26 GSD patients followed in our center for five years. We reported the new clinical findings and novel pathogenic variants that we observed in our study. To the best of our knowledge, early peripheral neuropathy after liver transplantation in GSD IV, and psychomotor retardation, seizure, autism signs and hypertrophic cardiomyopathy in GSD IXa, are the first to be reported in the literature. Hearing impairment in GSD I is also rarely reported.

In our study, two novel variants of *GYS2* associated with hepatic GSD0 [7,8] were detected in three patients; (c.1307A>C p.Gln436Pro) (P3, homozygous) and [c.607A>G p.(Thr203Ala)] (P1,2, heterozygous). Both siblings (P1,P2) had good blood-glucose control, with frequent protein-rich meals and nighttime feedings of uncooked cornstarch. They had normal growth and development. On the other hand, P3 had no clinical findings until the age of five, and was diagnosed with incidentally detected hypoglycemia. These two novel mutations might be related to a mild phenotype, and should be confirmed in further studies.

Another novel mutation of *G6PC* associated with GSDIa was detected in a patient; c.562+1G>A (P7, homozygous). The most striking finding was the bilateral sensorineural hearing impairment that was detected in two of our patients with different mutations (one with common, previously defined; c.247C>T and one with novel; c.562+1G>A, P6, P7, respectively) [9,10]. Iwanicka-Pronicka et al. [11] reported hearing impairment “at birth” in four (2 GSDIa; 2 GSDIb) out of 40 GSDI cases (20 patients with each subtype). The underlying mechanism has not been yet determined. Hearing impairment was determined when P6 was 6 months old and P7 was 18 months old; both of them passed newborn hearing screening tests and have normal brain MRI (magnetic resonance imaging), EEG (electroencephalogram), and neurocognitive development; they had a cochlear implant at the age of 1 and 2, respectively. Both have mild disarticulation and speech disturbance. As their hearing was normal at birth, auditory dysfunction gene panel or whole exome sequencing (WES) was not performed. For this reason, it would be appropriate to perform hearing evaluation in order to detect hearing loss early in GSD type 1 patients. Short stature and osteoporosis are remarkable findings among patients at any age, and may be due to inappropriate metabolic control, poor nutrition, the effects of lactic acidosis, or accompanying endocrinological problems (hypogonadism) [12,13,14]. The occurrence of osteoporosis in all of our patients, except the younger (P5), can be attributed to the above-mentioned factors. However, the short stature was also determined at P5. Kidneys were affected in three cases (P6–P8). The size and echogenicity of both kidneys increased in grade 1. One case has microalbuminuria, and none of them have impaired renal function tests. Renal involvement seems to be a complication that develops over time in advanced ages. The first sign of kidney involvement can be detected on ultrasound before clinical signs develop.

In our three patients, previously reported mutations were detected in the GSDIII-related *ALG* gene (P10–12) (Table 3) [15,16]. While all of the cases had liver and muscle involvement, heart involvement was not observed during the follow-up.

Two missense mutations were identified in *GBE1*-related GSDIV (one previously defined; c.1492G>A p.E498K and one novel; c.1054G>C p.Asp352His, P13, P14, respectively). P14 typically presented with hypotonia, myopathy, and hepatopathy. There is no treatment for this case other than liver transplantation [17]. Apart from that, it also causes a complex neurological condition called “Adult Polyglucosan Body Disease” (APBD), which shows symptoms after the fifth decade of life. It presents a variable combination of cognitive impairment, pyramidal tetraparesis, peripheral neuropathy, cerebellar dysfunction, and extrapyramidal signs [18,19]. Our P13 patient had mild developmental delay and peripheral sensory neuropathy at the age of six, which is an interesting early finding. To the best of our knowledge, there is no reported case in the literature that has both severe hepatic and neuromuscular involvement at this age. Performing neurological follow-up of patients with *GBE1* mutation from an earlier age will provide a chance for early detection of neurological findings.

We found a novel pathogenic variation in *PYMG* -related GSDV; c.772+2_772+3delTG (P16, homozygous). P17 had a previously reported mutation (Table 3). GSDV leads to exercise-induced myalgia and recurrent myoglobinuria, which may result in acute renal failure. No rhabdomyolysis attack was observed in either of our patients with appropriate treatment.

Three novel pathogenic *PYGL* mutations related GSDVI were identified in four patients; c.1355G>T (p.Gly452Val) (P17, homozygous), c.2380-1G>C p.(?) (P18, P19, homozygous) and c.921_924del (p.His308Leufs*8) (P20, homozygous). GSDVI may present with different combinations of findings including hepatomegaly, mild-to-moderate hypoglycemia, hyperlactatemia, hyper-transaminasemia, and short stature [20,21]. Short stature with normal neuromotor development should be a warning sign for GSD type VI. All four patients achieved a normal growth rate with appropriate treatment. All the clinical signs and biochemical parameters were improved.

GSDIX is a group of glycogenoses caused by hepatic phosphorylase kinase deficiency, a hexadecameric enzyme comprising four copies each of four unique subunits encoded by four different genes; *PHKA1*, *PHKA2*, *PHKB*, and *PHKG2* [20,22]. Five out of six patients had novel mutated genes as follows; in *PHKA2* c.1978C>T (p.Leu660Phe) (P22, homozygous) c.3028-2A>G (p.?) (P23, homozygous), c.3201G>T (p.Trp1067Cys) (P24, homozygous), in *PHKB* exon 18_21 deletion (p.?) (P25, homozygous), in *PHKA1* c.1963C>T (p.Arg655Cys) (P26, homozygous). The most common findings are hepatomegaly, short stature, delay in motor development, the elevation of transaminases, cholesterol, and triglyceride, fasting hyperketosis, and hypoglycemia. Neurological involvement is only in the form of mild motor-development delay in the GSDIX phenotype (6). Conspicuously, the clinical presentation in our GSDIX group was different from the literature. Unexpected new findings in our patients were marked psychomotor retardation (P22, P23, P24), seizure (P22, P24), autism signs (P22) and hypertrophic cardiomyopathy (P23). Therefore, GSD was not considered according to clinical presentation at admission, and exome sequencing was performed instead of the GSD panel test. The diagnoses of these patients were achieved by CES because of these different presentations. No additional pathologic variants were found in bioinformatic analyses to explain these findings.

## 5. Conclusions

This study identifies 12 novel mutations as well as diverse and new clinical features in GSD patients. It seems difficult to establish phenotype–genotype correlations in all types of GSDIX. To the best of our knowledge, hearing impairment in GSD I, early peripheral neuropathy after liver transplantation in GSD IV, and psychomotor retardation, seizure, autism signs and hypertrophic cardiomyopathy in GSD IXa can be considered as newly determined, rare and unexcepted findings.

## 6. Limitations of the Study

Although there is a high number of 26 patients in terms of general glycogen storage diseases for one single center, the small size of GSDs’ subgroups is the limitation of the study in terms of interpretation.

## Figures and Tables

**Table 1:** Demographic and clinical findings of the patients.

ID	GSD Type	Gender	Onset (Month)	Follow-Up Time (Year)	Hypoglycemia	Liver	Muscle	Hearth	Kidney	Short Structure	Mental Retardation	Additional Finding
P1	GSD0	F	4	16	+	−	−	−	−	−	−	−
P2	GSD0	M	6	7	+	−	−	−	−	−	−	−
P3	GSD0	F	37	3	+	−	−	−	−	−	−	−
P4	GSDIa	F	2	5	+	+	−	−	−	+	−	osteoporosis
P5	GSDIa	M	1	4	+	+	−	−	−	+	−	congenital hypothyiroidism
P6	GSDIa	F	4	5	+	+	−	−	+	+	−	hearing loss, osteoporosis
P7	GSDIa	M	3	14	+	+	−	−	+	+	−	hearing loss, osteoporosis
P8	GSDIa	M	1	11	+	+	−	−	+	+	−	osteoporosis
P9	GSDIb	M	1	5	+	+	−	−	−	+	−	neutropenia, aphthous stomatitis
P10	GSDIII	F	2	4	+	+	+	−	−	−	−	−
P11	GSDIII	F	12	1.5	+	+	+	−	−	−	−	−
P12	GSDIII	M	17	4	+	+	+	−	−	+	−	−
P13	GSDIV	F	9	7	−	+	−	−	−	−	+	liver tranplantation, neuropathy
P14	GSDIV	F	22	0.5	−	+	−	−	−	−	−	liver tranplantation
P15	GSDV	M	25	0.25	−	−	+	−	−	−	−	−
P16	GSDV	F	28	0.5	−	−	+	−	−	−	−	−
P17	GSDVI	M	4	5	+	+	+	−	−	+	−	−
P18	GSDVI	M	7	5	+	+	−	−	−	+	−	−
P19	GSDVI	F	7	5	+	+	−	−	−	+	−	−
P20	GSDVI	F	30	3	+	+	−	−	−	+	−	−
P21	GSDIXa	M	9	1.5	+	+	−	−	−	+	−	−
P22	GSDIXa	M	10	0.5	−	+	−	−	−	+	+	autism, seizure
P23	GSDIXa	M	19	0.5	−	+	−	+	−	+	+	−
P24	GSDIXa	M	11	2	−	+	−	−	−	−	+	seizure
P25	GSDIXb	M	15	2	−	+	+	−	−	+	−	−
P26	GSDIXd	M	26	1	−	+	+	−	−	−	−	thrombocytopenia

“+” (plus) means presence of the finding, “−” (minus) means absence of the finding.

**Table 2:** Initial laboratory and imaging features of patients.

ID	Type	Glycose mg/dL	AST U/L	ALT IU/L	CPK U/L	LDL mg/dL	HDL mg/dL	Cholesterol mg/dL	Triglyceride mg/dL	AFP ng/mL	Lactate mg/dL	Ketone	Ultrasound and/or ECHO
P1	GSD0	37	26	16	55	95	61	168	68	1	30	+	normal
P2	GSD0	35	30	18	68	79	68	172	56	1	26	++	normal
P3	GSD0	49	16	30	67	58	65	136	64	1	11	+	normal
P4	GSDIa	12	134	73	57	126	27	323	2007	2	65	+	grade 1 steatosis
P5	GSDIa	14	65	71	49	136	41	213	546	2	40	++	hepatomegaly
P6	GSDIa	9	282	133	67	149	23	341	1158	1	53	+++	heterogeneity in liver and kidney
P7	GSDIa	16	257	305	97	171	13	304	1200	2	97	+	heterogeneity in liver and kidney
P8	GSDIa	73	197	187	67	184	44	284	944	1.5	35	neg	hepatomegaly, heterogenity
P9	GSDIb	20	62	45	76	94	19	167	267	1.2	26	neg	grade 1 steatosis
P10	GSD III	34	226	327	1760	138	12	225	370	1	43	neg	grade 2 steatosis hepatomegaly
P11	GSDIII	71	705	867	867	186	30	217	479	4.4	42	neg	grade 1 steatosis, hepatomegaly
P12	GSDIII	23	160	123	424	145	8	234	471	41	25	+	grade 1 heterogeneity, hepatomegaly
P13	GSDIV	74	317	137	54	96	28	139	75	13	12	neg	nodular heterogeneous liver
P14	GSDIV	65	158	14	57	104	46	172	106	18	13	neg	nodular heterogeneous in liver
P15	GSDV	86	50	29	556	78	45	139	69	0.8	15	neg	normal
P16	GSDV	100	33	18	318	95	38	178	69	−	−	neg	normal
P17	GSDVI	74	545	444	87	104	22	178	241	2	25	+	hepatomegaly
P18	GSDVI	48	193	78	69	72	29	124	115	1.9	26	++	hepatomegaly
P19	GSDVI	39	60	64	98	94	36	158	138	2.7	31	+	hepatomegaly
P20	GSDVI	59	74	78	89	112	39	125	67	1.7	12	+	grade 1 steatosis, hepatomegaly
P21	GSDIXa	45	225	189	356	153	17	224	276	1	12	+	hepatomegaly, grade 1 steatosis
P22	GSDIXa	56	199	111	525	107	33	159	98	2	38	++	hepatomegaly
P23	GSDIXa	65	86	98	215	164	45	198	218	1.2	15	neg	hepatomagaly, heterogenity, hypertophic CMP
P24	GSDIXa	75	68	64	218	120	72	208	71	−	26	neg	hepatomagaly
P25	GSDIXb	78	74	50	562	89	61	169	76	5	11	++	grade 1 heterogeneity
P26	GSDIXd	67	80	84	292	76	48	138	67	1	9	+++	hepatomegaly

Abbreviations: AST: Aspartate transaminase, ALT: Alanine transaminase, CPK: Creatine phosphokinase, LDL: Low-density lipoprotein-cholesterol, HDL: High-density lipoprotein-cholesterol, AFP: Alpha-fetoprotein, ECHO: echocardiography. “+” (plus) means presence of the finding, “−” (minus) means absence of the finding.

**Table 3:** Molecular assays of patients.

ID	Type	Gene	Inheritence	Allele 1	Allele 2
P1	GSD0	*GYS2*	AR	**c.607A>G p.Thr203Ala**	c.1145G>A p.(Gly382Glu)
P2	GSD0	*GYS2*	AR	**c.607A>G p.Thr203Ala**	c.1145G>A p.(Gly382Glu)
P3	GSD0	*GYS2*	AR	**c.1307A>C p.Gln436Pro**	**c.1307A>C p.Gln436Pro**
P4	GSDIa	*G6PC*	AR	c.247C>T p.R83C	c.247C>T p.R83C
P5	GSDIa	*G6PC*	AR	c.247C>T p.R83C	c.247C>T p.R83C
P6	GSDIa	*G6PC*	AR	**c.562+1G>A**	**c.562+1G>A**
P7	GSDIa	*G6PC*	AR	c.247C>T p.Arg83Cys	c.247C>T p.Arg83Cys
P8	GSD1a	*G6PC*	AR	c.247C>T p.Arg83cys	c.247C>T p.Arg83cys
P9	GSDIb	*SLC37A4*	AR	c.1043_1044delCT p.Pro348ArgfsTer5	c.1043_1044delCT p.Pro348ArgfsTer5
P10	GSD III	*AGL*	AR	c.1019delA p.Gln340fs	c.1019delA p.Gln340fs
P11	GSDIII	*AGL*	AR	c.1020del p.Glu340Aspfs*9	c.1020del p.Glu340Aspfs*9
P12	GSDIII	*AGL*	AR	c.4126C>T p.Gln1376	c.4126C>T p.Gln1376
P13	GSDIV	*GBE1*	AR	c.1492G>A p.Glu498Lys	c.1492G>A p.Glu498Lys
P14	GSDIV	*GBE1*	AR	**c.1054G>C p.Asp352His**	**c.1054G>C p.Asp352His**
P15	GSDV	*PYGM*	AR	c.1A>G p.Met1Val	c.1A>G p.Met1Val
P16	GSDV	*PYGM*	AR	**c.772+2_772+3delTG**	**c.772+2_772+3delTG**
P17	GSDVI	*PYGL*	AR	**c.1355G>T p.Gly452Val**	**c.1355G>T p.Gly452Val**
P18	GSDVI	*PYGL*	AR	**c.2380-1G>C IVS19_1G>C**	**c.2380-1G>C IVS19_1G>C**
P19	GSDVI	*PYGL*	AR	c.2380+1G>C IVS19+1G>C	c.2380+1G>C IVS19+1G>C
P20	GSDVI	*PYGL*	AR	c.921_924del p.His308Leufs*8	c.921_924del p.His308Leufs*8
P21	GSDIXa	*PH KA2*	XL	c.3614C>T p.Pro1205Leu	c.3614C>T p.Pro1205Leu
P22	GSDIXa	*PHKA2*	XL	**c.1978C>T p.Leu660Phe**	**c.1978C>T p.Leu660Phe**
P23	GSDIXa	*PHKA2*	XL	**c.3028-2A>G**	**c.3028-2A>G**
P24	GSDIXa	*PHKA2*	XL	**c.3201G>T p.Trp1067Cys**	**c.3201G>T p.Trp1067Cys**
P25	GSDIXb	*PHKB*	AR	**Exon18_21 deletion**	**Exon18_21 deletion**
P26	GSDIXd	*PHKA1*	XL	c.1963C>T p.Arg655Cys	c.1963C>T p.Arg655Cys

Abbreviations: AR: autosomal recessive, AD: autosomal dominant, XL: X-linked. The novel variants are shown in bold.

## Data Availability

The data presented in this study are available on request from the corresponding author. The data are not publicly available due to privacy.
